# CD8^+^T cell–specific induction of NKG2D receptor by doxorubicin plus interleukin-12 and its contribution to CD8^+^T cell accumulation in tumors

**DOI:** 10.1186/1476-4598-13-34

**Published:** 2014-02-24

**Authors:** Jiemiao Hu, Shiguo Zhu, Xueqing Xia, Liangfang Zhang, Eugenie S Kleinerman, Shulin Li

**Affiliations:** 1 Department of Pediatrics–Research, The University of Texas MD Anderson Cancer Center, Houston, Texas, USA; 2 School of Basic Medical Sciences, Shanghai University of Traditional Chinese Medicine, Shanghai, P.R. China; 3 Department of NanoEngineering, University of California, 9500 Gilman Drive, La Jolla, San Diego, CA 92093, USA; 4 Department of Pediatrics–Research, Unit 0853, The University of Texas MD Anderson Cancer Center, The University of Texas Graduate School of Biomedical Sciences at Houston, 1515 Holcombe Blvd, Houston, TX 77030, USA

**Keywords:** Interleukin-12, Doxorubicin, Tumor-infiltrating lymphocytes, NKG2D^+^CD8^+^T cells

## Abstract

**Background:**

Increased infiltration of CD8^+^T cells into tumors has a positive impact on survival. Our previous study showed that doxorubicin (Dox) plus interleukin-12 (IL-12) boosted the accumulation of CD8^+^T cells in tumors and had a greater antitumor effect than did either agent alone. The purpose of this study was to determine the impact of NKG2D expression on CD8^+^T cell infiltration and antitumor efficacy.

**Methods:**

Tumor-bearing mice were administered Dox, *IL-12* plasmid DNA, or both via intraperitoneal injection or intramuscular electroporation. The induction of NKG2D on CD8^+^T cells and other lymphocytes was analyzed via flow cytometry, and NKG2D-positive CD8^+^T cell–specific localization in tumors was determined by using immunofluorescence staining in various types of immune cell–depleted mice.

**Results:**

The combination of Dox plus IL-12 specifically increased expression of NKG2D in CD8^+^T cells but not in other types of immune cells, including NK cells, which naturally express NKG2D. This induced NKG2D expression in CD8^+^T cells was associated with increased accumulation of CD8^+^T cells in murine tumors. Administration of NKG2D-blocking antibody or CD8^+^T cell–depletion antibody abrogated the NKG2D^+^CD8^+^T cell detection in tumors, whereas administration of NK cell–depletion antibody had no effect. Increased NKG2D expression in CD8^+^T cells was associated with increased antitumor efficacy *in vivo*.

**Conclusion:**

We conclude that Dox plus IL-12 induces NKG2D in CD8^+^T cells *in vivo* and boosts NKG2D^+^CD8^+^T-dependent antitumor immune surveillance. This discovery reveals a novel mechanism for how chemoimmunotherapy synergistically promotes T cell–mediated antitumor immune surveillance.

## Background

Infiltration of immune effector cells, such as T cells, natural killer (NK) cells, and macrophages, into tumors is known to suppress tumor cell growth [1-3]. The number of tumor-infiltrating lymphocytes may serve as a valuable prognostic marker for immunotherapy in many cancers, including breast cancer, melanoma, non–small-cell lung cancer, and ovarian cancer [4-7].

Chemotherapeutic agents used to treat cancer are generally believed to directly kill tumor cells [8]; however, accumulating evidence suggests that chemotherapeutic agents facilitate infiltration of immune effector cells into tumors and thus sensitize tumor cells to immune cell attack [9]. In fact, chemotherapy-induced immune response may serve as a predictor of therapeutic outcome in cancer patients [10]. Supporting this view are several reports showing that some chemotherapeutic compounds exhibit a more promising antitumor effect in patients who have higher levels of tumor-infiltrating lymphocytes after treatment than in patients who have lower levels of these cells [11]. In our previous study, we found that the combination of doxorubicin (Dox) and interleukin-12 (IL-12) had much greater antitumor efficacy than did either agent alone in 4T1 tumor–bearing mice [12]. This increased antitumor efficacy was associated with a substantial increase in CD8^+^T cell infiltration at tumor sites, but the mechanism for this CD8^+^T cell accumulation at the tumor site is largely unknown.

Natural killer group 2, member D receptor (NKG2D)–dependent immune surveillance plays a key role in suppressing tumor progression [13-15]. NKG2D is a lectin-like receptor protein that is expressed on NK, NKT, γδT, and some αβCD8^+^ T cells [16,17]. NKG2D is detected on CD8^+^T cells in both humans and mice, in mice on *active* CD8^+^T cells only [18,19]. As an activating receptor, NKG2D regulates innate and adaptive immune responses against infections and cancers [20]. In melanoma patients, tumor-infiltrating NKG2D-positive T cells were shown to have promising antitumor efficacy [21]. In the mouse tumor microenvironment, NKG2D-positive CD8^+^T cells were critical in recognizing tumor cells for tumor immunosurveillance [22]. We reasoned that a therapeutic strategy that increases the expression of NKG2D receptor on CD8^+^T cells may contribute tumor infiltration. Treatment with IL-12 modestly enhanced NKG2D expression on NK cells *in vitro*[23], but the effects of IL-12 on NKG2D expression on NK cells and CD8^+^T cells *in vivo* are unknown.

Our purpose for this study was to determine whether Dox plus IL-12 induces NKG2D expression in T cells and whether accumulation of NKG2D-positive CD8^+^T cells in tumors is dependent on NKG2D induction. Our central hypothesis was that Dox enhances IL-12–mediated NKG2D expression on CD8^+^T cells and that this increased NKG2D expression facilitates the accumulation of CD8^+^T cells in tumors and therefore enhances the antitumor efficacy of this combination [12]. We have confirmed this hypothesis by using *in vivo* and *in vitro* approaches. This study for the first time reveals that Dox plus IL-12 increases expression of the NKG2D receptor in CD8^+^T cells, thereby increasing accumulation of NKG2D-positive CD8^+^T cells in tumors to promote antitumor immune surveillance.

## Results

### NKG2D was specifically induced on CD8^+^T cells by Dox plus IL-12 but not on other types of immune cells

**Figure 1:**
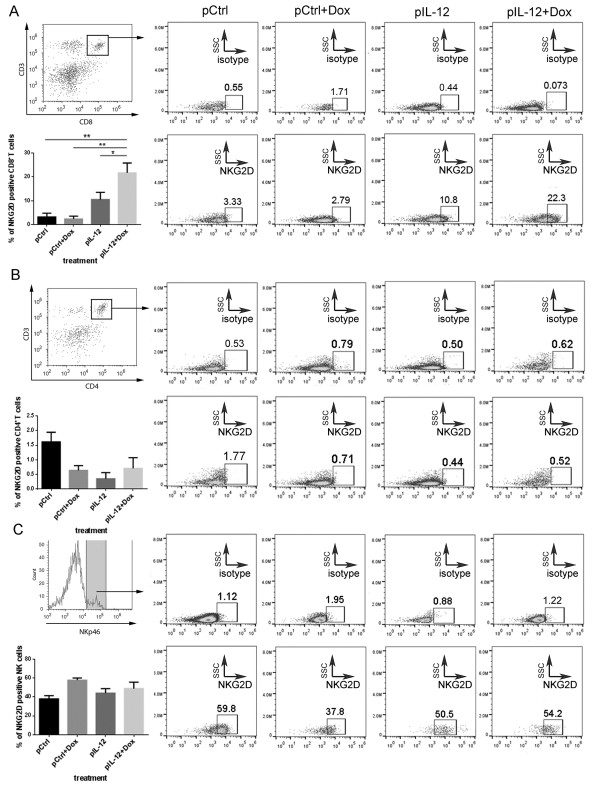
**CD8**^**+**^**T cell–specific induction of NKG2D by co-administration of Dox and IL-12.** Groups of tumor-bearing BALB/C mice were subjected to one of four standard treatments: control DNA (pCtrl), Dox plus control DNA (pCtrl + Dox), IL-12 (pIL-12), or Dox plus IL-12 (Dox + pIL-12) (n = 3 per treatment group). NKG2D expression was determined in CD8^+^T cells **(A)**, CD4^+^T cells **(B)**, and NK cells **(C). (A, B)** To measure NKG2D expression in CD8^+^T cells and CD4^+^T cells, splenocytes were stained with PE-Cy7 anti-mouse -NKG2D, PE anti-mouse CD3ϵ, and FITC anti-mouse CD8 or CD4 antibody or cognate isotype control antibody for detection of T cells, CD8^+^T cells, and CD4^+^T cells, respectively. CD3/CD8– or CD3/CD4–positive lymphocytes were gated with use of flow cytometry and then were further analyzed to determine their levels of NKG2D expression. **(C)** To measure NKG2D expression in NK cells, splenocytes were stained with PE-Cy7 anti-mouse NKG2D and FITC anti-mouse NKp46 or with corresponding isotype control antibody. NKp46-positive lymphocytes were selected by flow cytometry and then analyzed to identify NKG2D-positive cells. All of the bar graphs represent the percentage of NKG2D receptor–positive cell population, as mean ± SEM (n = 3; *P < 0.05, **P < 0.01, ***P < 0.001).

IL-12 modestly enhanced NKG2D expression on NK cells *in vitro*[23]. To determine the effects of Dox and IL-12 on NKG2D expression, the levels of NKG2D expression in various populations of lymphocytes from 4T1 tumor–bearing mice treated with control DNA, Dox plus control DNA, *IL-12* DNA alone, or Dox plus *IL-12* DNA were compared. Splenocytes from the mice receiving one of the above four treatments were stained with antibodies that detect NKG2D, CD4^+^T, CD8^+^T, and NK cells and analyzed via flow cytometry. Previously published results showed that NKG2D is constitutively expressed on NK and activated CD8^+^T cells [16,17,24]. In our study, NKG2D expression was significantly increased only on CD8^+^T cells, primarily in the mice treated with Dox plus IL-12 (Figure 1A*-*C). IL-12 seemed to reduce the NKG2D-positive CD4^+^T population (Figure 1B) and had little effect on the NKG2D-positive NK cell population (Figure 1C).

**Figure 2:**
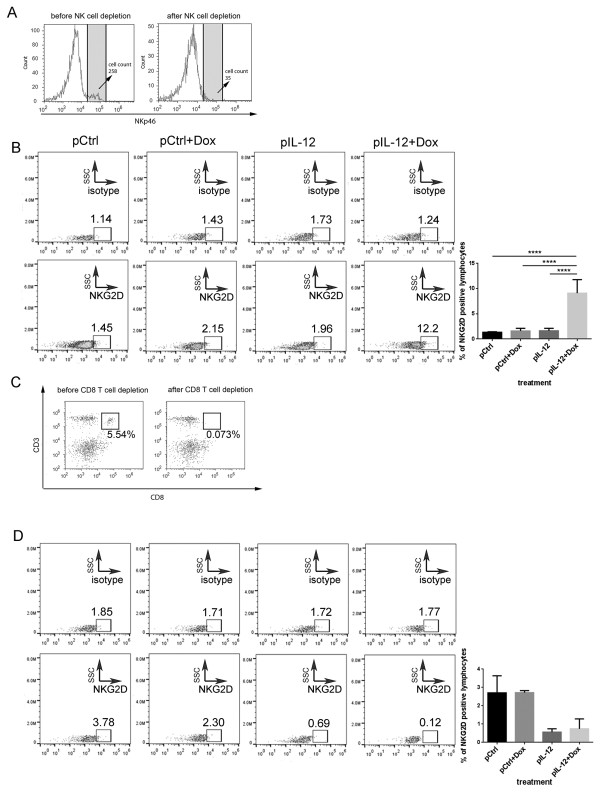
**NKG2D expression in NK cell–depleted and CD8**^**+**^**T cell–depleted tumor-bearing mice.** Groups of tumor-bearing BALB/C mice received one of the four standard treatments (control DNA, Dox plus control DNA, IL-12, or Dox plus IL-12; n = 3 per treatment) and also were subjected to NK cell or CD8^+^T cell depletion. **(A)** Efficiency of NK cell depletion. Splenocytes were collected and stained with NKp46 to confirm NK cell depletion. **(B)** NKG2D induction in total lymphocytes after NK cell depletion. Splenocytes were collected and stained with anti-mouse PE-Cy7 NKG2D or isotype control antibody, and the level of NKG2D induction was determined using flow cytometry. **(C)** The efficiency of CD8^+^T cell depletion. Splenocytes were collected and stained with PE CD3ϵ and FITC CD8 antibodies to confirm CD8^+^T cell depletion. **(D)** NKG2D expression in total lymphocytes by the indicated treatments after CD8^+^T cell depletion. Splenocytes were collected and stained with PE-Cy7 anti-mouse NKG2D or isotype control antibody, and the level of NKG2D was determined by using flow cytometry. All of the bar graphs represent the percentage of NKG2D receptor–positive cell population, as mean ± SEM (n = 3, ****P < 0.0001).

To validate the finding that Dox plus IL-12 induces NKG2D expression on the CD8^+^T cell population but not on other types of immune cells, we determined NKG2D expression in mice depleted of NK or CD8^+^T cells by administering a depleting antibody (Figure 2). The working hypothesis was that depletion of CD8^+^T cells would eliminate NKG2D expression in splenocytes if CD8^+^T cells were the sole cell population in which NKG2D was induced by Dox plus IL-12. To test this hypothesis, NK or CD8^+^T cell–depleted mice were treated with one of the four standard treatments described above. The splenocytes collected from these mice were subjected to the same flow cytometry analysis as described for wild-type mice (Figure 1). As shown in Figure 2A and C, NK cell–depletion antibody and CD8^+^T cell–depletion antibody effectively removed NK and CD8^+^T cells, respectively. In the NK cell–depleted mice, induction of NKG2D by Dox plus IL-12 was detected on total lymphocytes (Figure 2B), consistent with the observation in the wild-type mice in which NK cells were present (Figure 1A). Dox plus IL-12 did not induce any NKG2D on lymphocytes in the CD8^+^T cell–depleted mice (Figure 2D). These results validated the conclusion that Dox plus IL-12 treatment specifically induced NKG2D expression in CD8^+^T cells.

### Induction of NKG2D expression by Dox plus IL-12 enhanced immune cell localization in tumor sites

We previously published results showing that Dox plus IL-12 increased tumor infiltration by CD8^+^T cells [12]. We hypothesized that this increase may have been associated with the induction of NKG2D on CD8^+^T cells reported here. Key to testing this hypothesis was determining whether the increased infiltration of immune cells into tumors was dependent on the expression of NKG2D on the immune cells. To provide such evidence, we first determined whether the number of NKG2D-positive immune cells was increased in tumors. Since only Dox plus IL-12 induced a high level of NKG2D-positive CD8^+^T cells (Figures 1 and 2), we expected that an increased level of NKG2D expression would be detected only in tumors of mice receiving Dox plus IL-12, not in tumors of mice receiving any of the other treatments (control DNA, Dox plus control DNA, IL-12).

**Figure 3:**
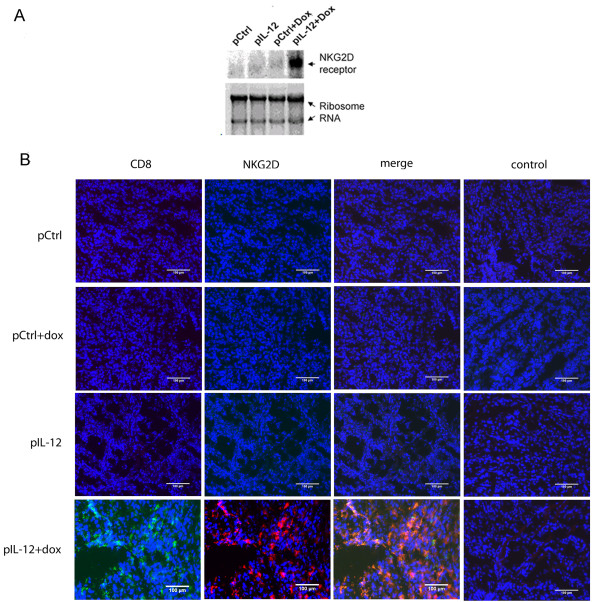
**NKG2D-dependent infiltration of CD8**^**+**^**T cells into tumors.** Tumors were collected from mice that had received one of the four standard treatments: control DNA, Dox plus control DNA, IL-12, Dox plus IL-12 (n = 3 per treatment group). **(A)** Infiltration of NKG2D-positive cells into tumors. Northern blot analysis was performed to detect *NKG2D* expression in tumors. Ribosomal RNA was used to confirm equal loading among samples. **(B)** NKG2D/CD8–positive cells in tumor sections by treatment received. Frozen tumor sections were stained with biotin anti-mouse NKG2D, anti-mouse CD8, or corresponding isotype control antibodies, then with streptavidin-conjugated Alexa fluor 594 or Alexa fluor 488 secondary antibodies. Data shown are representative of three independent experiments. The scale bar is equivalent to 100 μm.

To quantitate the infiltration of NKG2D-positive immune cells into tumors, we analyzed the levels of *NKG2D* mRNA in the tumors by Northern blotting. Since tumor cells do not express *NKG2D*, any detected level of *NKG2D* expression could be attributed to tumor-infiltrating immune cells. As expected, a high level of *NKG2D* expression was detected only in the tumors of mice treated with Dox plus IL-12 (Figure 3A). To validate the Northern blotting result, we performed colocalization analyses of NKG2D and CD8 in tumor sections *via* immunofluorescence staining. In this analysis, a high number of NKG2D/CD8–positive immune cells were detected and colocalized in tumors of mice receiving Dox plus IL-12 but not in tumors of mice receiving any other treatment (Figure 3B). The NKG2D signal could not be colocalized with CD4 (Additional file 1: Figure S1A) or NK marker NKp46 (Additional file 1: Figure S1B). In fact, neither CD4^+^ nor NK cells were detectable in any tumors (Additional file 1: Figure S1A and S1B). This result is consistent with the lack of NKG2D induction in both CD4^+^ and NK cells shown in Figure 1. The inability to detect CD4^+^ and NK cells was not due to defective antibodies because these antibodies were able to detect the cognate cells in splenocytes (data not shown).

**Figure 4:**
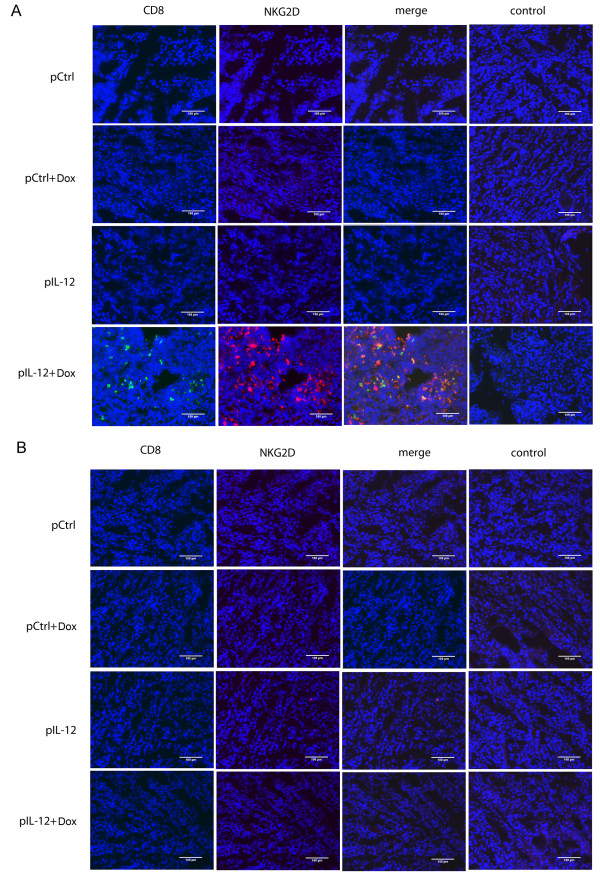
**The effect of NK cell or CD8**^**+**^**T cell depletion on NKG2D-positive lymphocyte localization in tumors.** Groups of mice (n = 3 per treatment group) were subjected to one of the four standard treatments: control DNA, Dox plus control DNA, IL-12, Dox plus IL-12. Tumor sections were stained as detailed in the Figure 3 legend. **(A)** Detection of NKG2D-positive cells in tumors from NK cell–depleted mice, **(B)** Absence of NKG2D-positive cells in CD8^+^T cell–depleted mice. Data shown are representative of three independent experiments. The scale bar is equivalent to 100 μm.

To confirm that the cells positive for both NKG2D and CD8 detected in tumors (Figure 3B) were CD8^+^T cells, the same immune cell depletion approach portrayed in Figure 2 was used. The rationale was that depletion of CD8^+^T cells would eliminate detectable NKG2D/CD8–positive cells in tumor tissues, whereas depletion of NK cells would not affect these signals if CD8^+^T cells were the true and sole population of cells in which Dox plus IL-12 induced NKG2D at a high level. As expected, we detected a high number of NKG2D/CD8–positive cells in tumors from NK cell–depleted mice that received Dox plus IL-12 (Figure 4A) but detected none in CD8^+^T cell–depleted mice (Figure 4B). These results clearly confirmed that CD8^+^T cells, but not NK cells, were the true NKG2D-positive cells detected in tumors.

### CD8^+^T cell localization in tumors was dependent on NKG2D

To determine whether NKG2D is crucial for CD8^+^T cell accumulation in tumors, 4T1 tumor–bearing mice were treated with Dox plus IL-12 plus control IgG or NKG2D-blocking antibody. This NKG2D blocking antibody C7 had been shown by other investigators to block NKG2D’s biological function [25,26]. Because this blocking antibody blocks NKG2D engagement to its ligand without depleting the blocked cells, Dox plus IL-12 treatment should still induce NKG2D-positive CD8^+^T cells. If NKG2D is crucial for CD8^+^T cell accumulation in tumors, then blocking NKG2D might reduce or impair the localization of CD8^+^T cells in tumors without reducing the NKG2D-positive CD8^+^T cells in spleens.

**Figure 5:**
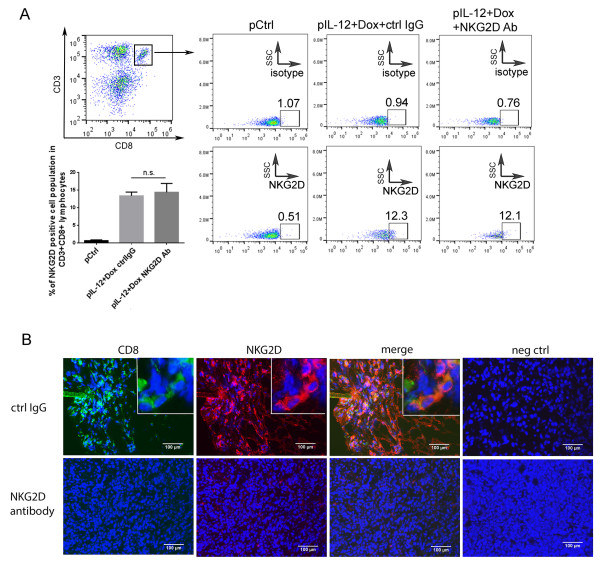
**The effect of NKG2D-blocking antibody on NKG2D-positive CD8**^**+**^**T cells and CD8**^**+**^**T cell accumulation in tumors.** 4T-1 tumor–bearing mice were subjected to one of two treatments: Dox plus IL-12 plus control IgG or Dox plus IL-12 plus NKG2D-blocking antibody (n = 3 per treatment). **(A)** NKG2D expression in CD8^+^T cells was determined as described for Figure 1 (n.s., not significant). **(B)** The presence of NKG2D/CD8–positive cells in tumor sections after the indicated treatments were determined as described for Figure 3.

As expected, blocking antibody did not reduce the number of NKG2D-positive CD8^+^T cells after Dox plus IL-12 treatment because an equal percentage of NKG2D-positive cells were detected in CD8^+^T cells in either the absence or the presence of the NKG2D-blocking antibody after this Dox plus IL-12 combination therapy (Figure 5A). However, CD8^+^T cells failed to localize into tumors in the presence of NKG2D-blocking antibody (Figure 5B), showing that NKG2D is required for CD8^+^T cell accumulation in tumors.

### Inhibition of tumor growth and metastasis by Dox plus IL-12 was dependent on NKG2D

**Figure 6:**
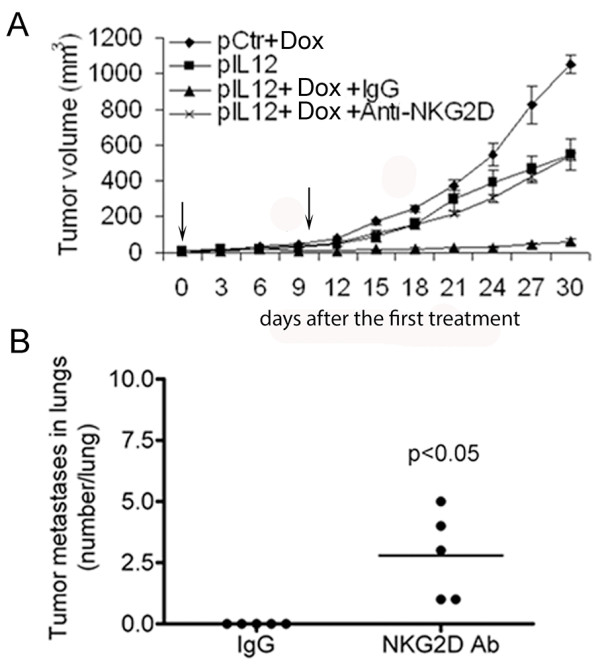
**Association between level of NKG2D expression on splenocytes and antitumor efficacy *in vivo. *(A, B)** Groups of tumor-bearing BALB/C mice (n = 5 per group) were subjected to one of the four standard treatments. Two independent experiments were performed. **(A)** Administration of anti-NKG2D antibody reduced Dox plus IL-12–mediated inhibition of tumor growth. Anti-NKG2D and IgG represent NKG2D-blocking antibody (clone C7) and control IgG antibody, respectively. The curves represent the tumor volumes as mean ± SEM. **(B)** Administration of anti-NKG2D antibody reduced Dox plus IL-12–mediated inhibition of metastatic tumor growth. The graph represents the numbers of lung metastases as the mean of number per lung ± SEM.

Finally, to determine whether the increase in NKG2D expression induced by Dox plus IL-12 accounted for the previously observed antitumor efficacy of this combination *in vivo*[12], we administered the NKG2D-blocking antibody to mice treated with Dox plus IL-12. Although control IgG did not affect the Dox plus IL-12–mediated inhibition of tumor growth, the NKG2D-blocking antibody completely reversed Dox plus IL-12–mediated inhibition of tumor growth (Figure 6A).

To further demonstrate the dependence of the antitumor activity of Dox plus IL-12 on NKG2D, we compared the numbers of spontaneous metastatic lung nodules in mice treated with Dox plus IL-12 along with anti-NKG2D antibody or control IgG. Dox plus IL-12 was given on day 7 and day 17 after tumor cell inoculation, and the mice were euthanized via CO_2_ inhalation 30 days after the first treatment. The findings were consistent with those of the tumor growth inhibition study shown in Figure 6A: treatment with the NKG2D-depleting antibody abrogated the Dox plus IL-12–mediated inhibition of spontaneous metastatic tumor growth (Figure 6B). This result supports the hypothesis that tumor growth inhibition mediated by Dox plus IL-12 is dependent on NKG2D upregulation.

## Discussion

Our results show that treatment with Dox plus IL-12 increased the number of NKG2D-positive CD8^+^T cells in tumor-bearing mice (Figures 1 and 2) and promoted the localization of NKG2D-positive CD8^+^T cells in tumors (Figures 3, 4, 5). This combination treatment also increased NKG2D-dependent antitumor efficacy *in vivo* (Figure 6).

Several known mechanisms account for IL-12–mediated antitumor efficacy, including induction of IFN-γ, promotion of type 1 helper T cell response, and stimulation of CD8^+^T cell antitumor response [27-29]. Likewise, Dox has been shown to act through several mechanisms, including disruption of DNA synthesis, initiation of DNA damage, and compromise of the cell membrane, to cause cell apoptosis [30]. This study revealed that NKG2D induction on CD8^+^T cells serves as an important mechanism for Dox-augmented IL-12–mediated tumor growth inhibition because the increased NKG2D expression on CD8^+^T cells plays a role in tumor-specific localization (Figures 3 and 5).

The dependence of antitumor efficacy on NKG2D was not surprising because others have discovered that silencing NKG2D, or its associated molecules DAP10 or DAP12, reduces the cytotoxicity of activated CD8^+^T cells and NK cells *in vitro*[31]. The surprising observation from this study is the CD8^+^T cell–specific induction of NKG2D by treatment with Dox plus IL-12 (Figure 1A). This is surprising because NKG2D is constitutively expressed on NK cells, and others found that IL-12 recombinant protein induces modest NKG2D expression on NK cells *in vitro*[23]. In contrast, our *in vivo* results showed that Dox plus IL-12 treatment expanded the NKG2D-positive CD8^+^T cell population (Figure 1A) but failed to change the NKG2D-positive NK cell population (Figure 1A). This observation was validated in NK cell–depletion and T cell–depletion studies (Figure 2).

*In vitro* results from others found that IL-12 induces modest NKG2D expression in NK cells [23], but our *in vivo* results could not confirm this observation. Instead, we found that NKG2D is induced in CD8^+^T cells. This observation may not be easily accepted but has been carefully validated in our study. The discrepancy between our observations and those of other authors is most likely due to the differences in model systems. Other investigators used an *in vitro* system to observe the modest NKG2D increases in NK cells, whereas we used an *in vivo* system; others used IL-12 alone, whereas we used Dox plus IL-12; finally, others used recombinant IL-12 protein, whereas we used an *IL-12* gene therapy approach, which yields only a very low level of IL-12 expression. In future study, we will explore the mechanism by which IL-12 plus Dox induces NKG2D *in vivo.* Finding this mechanism will not be easy, based on our preliminary study, in which direct treatment of splenocytes with IL-12 plus Dox did not induce NKG2D.

It is still a puzzle how NKG2D-positive CD8^+^T cells accumulate in tumors after IL-12 plus Dox treatment. Markiewicz *et al.*[32] indicated that NKG2D ligand Rae-1 expression recruits NKG2D-positive cytotoxic T cells independent of antigen recognition. NKG2D ligands are primarily present in tumor cells or infected cells but not in normal tissues [17,33,34]. Recently, NKG2D ligand Rae-1 expression was also observed in tumor vasculature [35]. These discoveries shed light on the possibility that NKG2D ligand expression in tumor cells or tumor vasculature attracts NKG2D-positive CD8^+^T cell accumulation. Moreover, it is known that chemokines affect the migration of lymphocytes to tumors. An earlier study stated that the expression of CCR5 and CXCR3 has a positive correlation with the accumulation of CD8^+^ and CD4^+^ T lymphocytes in invasive colorectal tumors [36]. Another study demonstrated that chemokine CCL2 and its receptor CCR2 are needed for human Vδ1T cell infiltration into various tumors including lung, prostate, liver, or breast cancers [37]. Moreover, CXCL10 was found to enhance tumoral lymphocytic infiltrate in multiple cancers. Mulligan *et al.*[38] indicated that in breast cancers, CXCL10 expression showed significant association with tumor-infiltrating CD4^+^ and CD8^+^ lymphocytes. Also, induction of CXCL10 and CCL5 in colorectal tumors by hyperactivated NF-κB selectively promoted the accumulation of T effector lymphocytes but reduced the T regulatory lymphocytes [39]. Therefore, IL-12 plus Dox treatment possibly reprograms chemokine expression in the tumor microenvironment, which boosts the NKG2D induction-associated recruitment of NKG2D-positive CD8+ T lymphocytes.

Other investigators found that a modest dose of Dox had the potential to boost immune response and potentiate the IL-2 effect against tumor cells [40]. In fact, one report demonstrated that the Dox-mediated therapeutic effect against cancer requires CD8^+^T cells and IFN-γ [41]. Although the mechanism was unknown in both cases, we speculate that the immune response may be boosted by upregulating NKG2D through a combination of Dox plus IL-2 or Dox plus IFN-γ.

## Conclusions

In summary, we have presented *in vivo* evidence that Dox plus IL-12 induces CD8^+^T cell–specific NKG2D induction, which facilitates the accumulation of NKG2D-expressing CD8^+^T cells in tumor sites. Others have found that induction of NKG2D ligands boosts NKG2D-mediated tumor cell death [17,24,26,42,43]. We expect that developing a strategy to simultaneously boost induction of the NKG2D ligand in tumors and NKG2D expression in immune cells, which will be the focus of our future effort, will greatly enhance the antitumor immune response and the treatment’s antitumor efficacy.

Finally, it is still not clear why NKG2D-positive NK cells fail to accumulate in tumors whereas NKG2D-positive CD8^+^T cells do accumulate. We speculate that independent engagement of another ligand and receptor between a tumor and CD8^+^T cells is required, a theory that we are currently investigating.

## Materials and methods

### Ethics statement

The mice used in this study were maintained under National Institutes of Health guidelines and according to procedures approved by the Institutional Animal Care and Use Committee of The University of Texas MD Anderson Cancer Center.

### *IL-12* gene construct and Dox

The mice were treated with a combination regimen of Dox plus an *IL-12* gene construct. The *IL-12* gene construct was obtained from Valentis, Inc. (Vilnius, Lithuania); the backbone of this construct was described in a previous publication [44]. The control plasmid DNA consisted of the same construct with the *IL-12* gene deleted. Plasmid DNA was prepared by using the endotoxin-free Mega preparation kit from Qiagen, Inc. (Valencia, CA) according to the manufacturer’s instructions. Doxorubicin (Bedford Laboratories, Bedford, OH) was purchased from the pharmacy at the Louisiana State University School of Veterinary Medicine.

### Tumor models and DNA delivery via intramuscular electroporation

Six- to eight-week-old female BALB/C mice weighing 18-20 g were obtained from the National Cancer Institute or Jackson Laboratory (Bar Harbor, ME) for this study. Murine invasive breast carcinoma 4T1 cells were maintained in Dulbecco modified essential medium containing 10% fetal bovine serum (Life Technologies, Grand Island, NY). Tumors were generated by subcutaneously inoculating BALB/C mice with 4T1 tumor cells (2×10^5^) suspended in a 30-μL volume of phosphate-buffered saline solution (PBS). Tumor dimensions were measured with calipers every 3 days, and tumor volume was calculated with use of the following formula: V = (π/8) *a* × *b*^2^, where V = tumor volume in cubic centimeters, *a* = maximum tumor diameter, and *b* = diameter at 90° to *a*[45].

Using protocols described previously, we injected *IL-12*–encoding or control plasmid DNA into the two hindlimb tibialis muscles of each mouse via electroporation [46]. This procedure yielded a blood IL-12 level greater than 100 pg/mL [47]. The electroporation parameters for intramuscular injection, which had previously been identified as optimal, were set at 350 V/cm and 20 ms pulse duration for 2 pulses [46].

Mice received one of four standard treatments: control plasmid DNA alone (control DNA), Dox plus control plasmid DNA (Dox plus control DNA), *IL-12* plasmid DNA alone (IL-12), or Dox plus *IL-12* plasmid DNA (Dox plus IL-12). The treatments were administered twice on days 7 and 17 after tumor cell inoculation. For each round of treatment, each mouse received 5 μg of DNA for each muscle, for a total of 10 μg of DNA. The dose of each Dox treatment was 5 mg/kg, and the Dox was administered intraperitoneally. Dox was administered at the same time as the plasmid DNA. Mice were euthanized via CO_2_ inhalation 4 days after the second treatment (day 21) and their tissues subjected to the analyses described in subsequent sections.

### CD8^*+*^T/NK cell depletion *in vivo*

For immune cell–depletion experiments, CD8^+^T cell–depletion antibody (clone 2.43) or NK cell–depletion antibody (anti-Asialo GM1) was administered to deplete CD8^+^T cells or NK cells, respectively. Tumor-bearing BALB/C mice were inoculated intraperitoneally with one of the antibodies (50 μg of antibody in 50 μL PBS) on day 7 along with the first treatment. Injection of the cell-depleting antibody was repeated twice a week.

### Flow cytometry analysis for detecting NKG2D-positive immune cells

Spleens from treated mice were homogenized gently in a 40-μm nylon strainer, and red blood cells were subjected to lysis with Puregene red blood cell lysis solution (Gentra Systems, Minneapolis, MN). Spleen cells (50,000 cells/sample) were stained with various antibodies to identify immune cell types: PE-Cyanine7 (PE-Cy7)-conjugated anti-mouse NKG2D (clone CX5) or isotype control antibody (eBioscience, San Diego, CA); fluorescein isothiocyanate (FITC)–conjugated anti-mouse CD4 (clone GK1.5) or CD8a antibody (clone 53-6.7) or the isotype control antibody (Pharmingen, San Diego, CA); PE-conjugated anti-mouse CD3ϵ (clone 145-2C11) and FITC-conjugated anti-mouse NKp46 antibody (clone 29A1.4) or its isotype control antibody (eBioscience, San Diego, CA). NKp46 was recently identified as a NK cell marker [48]. Anti-NKG2D C7 antibody was generously provided by Dr. Wayne Yokoyama (Washington University School of Medicine). The stained cells were analyzed on an Attune acoustic focusing cytometer (Applied Biosystems, Inc., Carlsbad, CA). Data were analyzed by using Attune software (Applied Biosystems, Inc.) or FlowJo software (Ashland, OR).

### RNA isolation and Northern blot analysis of gene expression

RNA was isolated from tumors with TRIzol reagent (Invitrogen, Carlsbad, CA) as described previously [49]. The details of Northern blot analysis of gene expression were presented in a previous publication [50]. Briefly, total RNA (4 μg) was subjected to denaturing by agarose gel electrophoresis, and ribosomal RNA was stained with ethidium bromide to ensure equal loading of all samples. The Northern blot was scanned with use of a Molecular Imager (Bio-Rad, Hercules, CA). The signal intensity was normalized to the level of the total ribosomal RNA.

### Immunofluorescence staining analysis

Frozen tumor sections were fixed with cold acetone, acetone plus chloroform (1:1), and acetone. Tissue sections were blocked with blocking buffer (5% normal horse serum and 1% normal goat serum in PBS) and incubated with rat anti-mouse CD8α (clone YTS105.18, AbD Serotec, Raleigh, NC), rat anti-mouse CD4 (clone RM4-5, BD Pharmingen, San Jose, CA), or rat anti-mouse NKp46 antibody (clone 29A1.4, Biolegend, San Diego, CA) overnight at 4°C. The next day, tissue sections were blocked and incubated with goat anti-rat Alexa fluor 488 secondary antibody (Life Technologies, Grand Island, NY) for 1 hour at room temperature. Tissues were then blocked and incubated with second primary antibody NKG2D-biotin antibody (1:50; R&D Systems, Minneapolis, MN) overnight at 4°C and second secondary antibody streptavidin-conjugated Alexa fluor 594 (Life Technologies, Grand Island, NY) for 1 hour at room temperature. Rat IgG was used as the negative control. Nuclei were counterstained with Hoechst 33258 (1:10,000) (Life Technologies, Grand Island, NY). Tumor sections were mounted in antifade fluorescence mounting medium (Life Technologies, Grand Island, NY). Slides were visualized under the Nikon eclipse Ti fluorescence microscope (Nikon, Melville, NY) with use of appropriate filters (original magnification, 100×).

### Statistical analysis

Tumor volume, lung metastases, and flow cytometry analyses were the primary outcomes measured. We used the 2-sided Student *t*-test to compare results between two treatment groups or one-way ANOVA to compare results from more than two treatment groups. GraphPad Prism software (GraphPad Software, Inc., La Jolla, CA) was used to determine the *P* values, and *P* values < 0.05 were considered statistically significant.

## Abbreviations

NK: Natural killer; Dox: Doxorubicin; IL-12: Interleukin-12; pCtrl: Control plasmid DNA; pIL-12: *IL-12* plasmid DNA; NKG2D: Natural killer group 2, member D; PBS: Phosphate-buffered saline solution; FITC: Fluorescein isothiocyanate; siRNA: Small-interfering RNA; KO: Knockout; IFN: Interferon.

## Competing interest

The authors have declared that no conflict of interest exists.

## Author contributions

JH generated most of the data and figures; XX performed Northern blotting and PCR analysis; SZ was a contributor to Figure 6; EK, LZ, and SL were primary contributors to the experimental design, MS integration, and editing. All authors’ read and approved the final manuscript.

## Author information

Shulin Li, professor at the University of Texas Graduate School of Biomedical Science (UTGSBS) in Houston, Department of Pediatrics Research, Endowed Chair; Chair of Cellular Immune Response Committee for American Society of Gene and Cell Therapy. Dr. Li has co-authored articles published in the journals *Science*, *Immunity*, *Journal of Experimental Medicine*, *Molecular Cell*, *Journal of the National Cancer Institute*, and *Nature Reviews*.

Eugenie Kleinerman, professor at UTGSBS, Department of Pediatrics Research; head of Department of Pediatrics Research, MD Anderson Cancer Center; Charter of NIH Study Section. Dr. Kleinerman has published more than 100 cancer-related articles.

Liangfang Zhang, associated professor at the University of California San Diego, has created a physiological nanoparticle vehicle for tumor-targeted delivery of doxorubicin and other chemical agents. His recent publications have appeared in the *Proceedings of the National Academy of Sciences of the United States of America*, *Nanobiotechnology*, and *Nanomedicine and Nanoscale*.

## Supplementary Material

Additional file 1: Figure S1 No infiltrating NKG2D positive CD4^+^T or NK cells were observed in tumors. Tumors were collected from mice as described in Figure 3. Frozen tumor sections were stained with biotin anti-mouse NKG2D **(A, B)**, anti-mouse CD4 **(A)**, anti-mouse NKp46 **(B)**, or corresponding isotype control antibodies **(A, B)**, then with streptavidin-conjugated Alexa fluor 594 or Alexa fluor 488 secondary antibodies **(A, B)**. Data shown are representative of three independent experiments. The scale bar is equivalent to 100 μm. Click here for file
