# Supplementary figures and images for: CD8+T cell–specific induction of NKG2D receptor by doxorubicin plus interleukin-12 and its contribution to CD8+T cell accumulation in tumors
Source: Mol Cancer. 2014 Feb 24;13:34. doi: 10.1186/1476-4598-13-34 (PMC3938086; doi:10.1186/1476-4598-13-34)

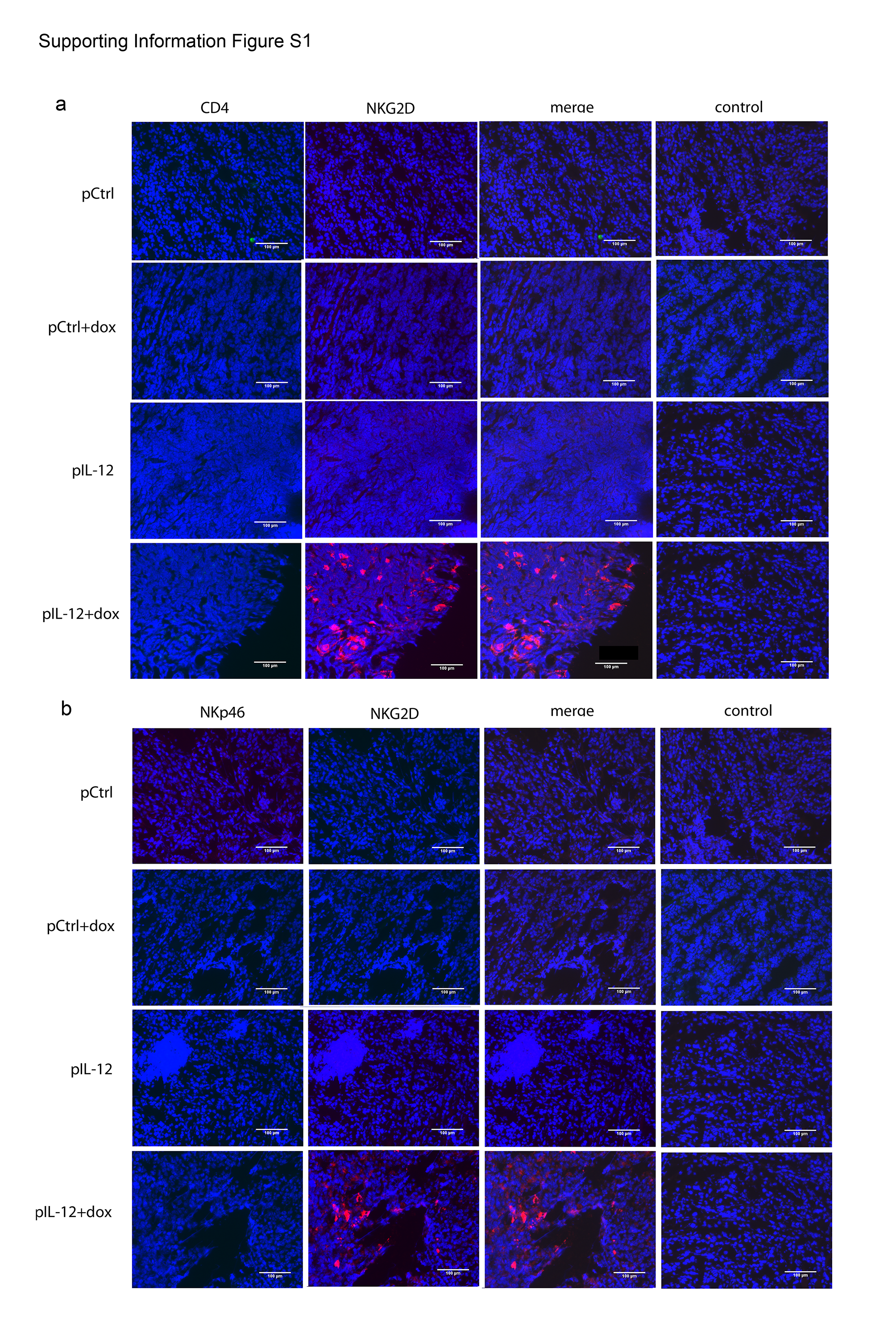

Supplement: Additional file 1: Figure S1 — No infiltrating NKG2D positive CD4+T or NK cells were observed in tumors. Tumors were collected from mice as described in Figure 3. Frozen tumor sections were stained with biotin anti-mouse NKG2D (A, B), anti-mouse CD4 (A), anti-mouse NKp46 (B), or corresponding isotype control antibodies (A, B), then with streptavidin-conjugated Alexa fluor 594 or Alexa fluor 488 secondary antibodies (A, B). Data shown are representative of three independent experiments. The scale bar is equivalent to 100 μm. [file 1476-4598-13-34-S1.tiff]
